# Are school-based violence prevention interventions inclusive and effective for children with disabilities? A systematic review of global evidence

**DOI:** 10.1016/j.eclinm.2024.103060

**Published:** 2025-01-17

**Authors:** Emily Eldred, Karen Devries, Anja Zinke-Allmang, Rizwana Mallick, Waliyah Mughis, Lena Morgon Banks, Amiya Bhatia

**Affiliations:** a Faculty of Epidemiology and Population Health, London School of Hygiene & Tropical Medicine, London, UK; b Department of Social Policy and Intervention, University of Oxford, Oxford, UK; c Children's Institute University of Cape Town, Cape Town, South Africa; d Department of Community Health Sciences and the Brain & Mind Institute, Aga Khan University, Karachi, Pakistan

**Keywords:** Disability, Violence against children, Randomised controlled trials, School-based interventions

## Abstract

**Background:**

Children with disabilities are twice as likely to experience violence compared to peers without disabilities. While evaluations of school-based interventions targeting the prevention of violence against children in schools are growing in number, it is unclear whether these interventions are inclusive of, or effective for, children with disabilities.

**Methods:**

We searched six databases (Medline, Cochrane Library, Embase, Global Health, PsycINFO, Web of Science) and utilised professional networks to identify systematic reviews which included randomised controlled trials (RCTs) of school-based violence prevention interventions up to May 2024. Once we identified our final sample of systematic reviews (n = 29) we hand searched the included papers within these reviews and included all RCTs of school-based violence prevention interventions. We applied criteria to assess disability inclusion and conducted a narrative synthesis of study characteristics, adaptations to intervention and/or data collection design, and effect estimates. We assessed risk of bias using the Cochrane Risk of Bias tool. This review was registered on PROSPERO (CRD42023463384).

**Findings:**

We identified 160 articles of school-based violence prevention interventions. Of these, 13 articles reporting on 10 trials (8.13%) explicitly mentioned disability: 3/10 trials reported on the magnitude of intervention effects among children with disabilities; 4/10 trials mentioned adaptations to research or intervention design to include children with disabilities; 6/10 trials mentioned disability as part of the sample characteristics but did not report further sub-group analysis. 3 trials were effective in reducing violence in schools for children with disabilities, with risk of bias ranging from ‘low’ (n = 1) to ‘some concerns’ (n = 2).

**Interpretation:**

Despite growing evidence on how to prevent school violence, there is limited research on the effect of such interventions for children with disabilities. There is a need for future evaluations to stratify effects by disability, conduct disability-inclusive research, and tailor interventions for children with disabilities.

**Funding:**

This research was partially funded by the 10.13039/501100020171Foreign, Commonwealth and Development Office under the PENDA project (PO8073).

**Research in context**

**Evidence before this study**

Every year 1 billion children experience violence across the world, impacting their short- and long-term health, wellbeing, and education. Schools provide an opportunity for early intervention to prevent violence due to their reach and role in a child's development. Several published systematic reviews have examined the effectiveness of school-based interventions to prevent violence in schools, including bullying, dating violence, corporal punishment and sexual abuse, finding that violence prevention in and around schools can be achieved through early intervention. Yet, there is less clarity on whether these interventions are inclusive of, or effective for, children with disabilities, who not only make up a large minority of children but are also at particular risk of experiencing violence compared to children without disabilities. Before starting this review, we searched electronic databases for any published papers and PROSPERO for any registered unpublished reviews examining school-based interventions to prevent violence against children with disabilities using randomised controlled trial methodology, which yielded no results.

**Added value of this study**

This review is the first synthesis of school-based violence prevention interventions that are inclusive of, or find an effect for, children with disabilities. We find that while randomised controlled trials evaluating school-based violence prevention exist, the majority do not mention children with disabilities. Of the few trials that specifically mentioned children with disabilities, only three conducted further analysis and four described adaptations to intervention or research design. The three trials that analysed the effect of school-based violence prevention interventions amongst children with disabilities, found that at least for some outcomes the intervention effects on violence reduction extended to children with disabilities. However, the variance in effectiveness of some outcomes suggests that interventions may need to adapt violence prevention efforts to be more inclusive of children with disabilities.

**Implications of all the available evidence**

To achieve violence prevention at scale and to meet global commitments such as the Sustainable Development Goals, it is important that future violence prevention efforts are inclusive of, and adapted for, children with disabilities. For researchers, this includes measuring disability status or functional difficulty in studies on violence, adapting data collection procedures from a disability justice lens, and powering trials for sub-group analysis by disability status. For practitioners, taking a ‘twin-track’ approach to design targeted interventions for children with disabilities, particularly for disability-targeted forms of violence, and adapting current interventions for children in mainstream schools for different types of disabilities is recommended.

## Introduction

Outside of the home, children spend most of their time at school.^1^ Schools are essential for children's education, social and emotional development, and can be an important context for early intervention in preventing violence.^2^ Schools can be sites of physical, emotional, and sexual violence from peers and school staff, including corporal punishment, bullying, relationship violence and sexual violence,^3^^,^^4^ which can influence a child's short-and long-term health and social outcomes.^5^, ^6^, ^7^, ^8^, ^9^, ^10^, ^11^, ^12^, ^13^ Violence victimisation occurs more frequently for children with disabilities, with global estimates suggesting children with disabilities are twice as likely to experience violence than their non-disabled peers^14^ and some evidence suggests children with disabilities are at higher risk of experiencing poly-victimisation compared to other forms of violence.^15^ Although children with disabilities are less likely to attend school in some countries,^16^ studies suggest that higher levels of violence victimisation extend into schools, as one study in Uganda found that children with disabilities (5.8%, n = 220 of sample) experienced higher levels of physical (69.2% vs 45.8%), sexual (3.9% vs 0.6%), and emotional violence (18.3% vs 8.2%) than children without disabilities.^17^ All violence against children is a violation of their rights, and the higher burden of violence among children with disabilities is unfair, unjust, and constitutes an inequity.^18^, ^19^, ^20^

Research on preventing violence within schools is growing and recent systematic reviews have found 69 school-based interventions focussing on reducing bullying,^21^, ^22^, ^23^ 68 to prevent dating violence,^24^, ^25^, ^26^, ^27^ 4 focussing on teacher violence,^28^ and 29 to prevent sexual abuse.^29^, ^30^, ^31^ Although children with disabilities will be included in these universally implemented school-based interventions, there is a dearth of evidence documenting whether interventions were adapted for, and effective for, children with disabilities. A review in 2014 on interventions to prevent violence against adults and children with disabilities found only 10 studies, and no studies in a school setting or a low-and-middle income country.^32^

Within public health trials and other research designs, as well as in interventions, children with disabilities are frequently invisible or excluded in several ways.^33^, ^34^, ^35^, ^36^ First, disability can be an explicit criterion for exclusion in research. Second, disability status may not be measured, or not adequately measured, in research. Third, inaccessible data collection procedures, such as not accounting for diverse communication needs in interviews, can also preclude children with disabilities from participation even if they are not explicitly excluded. Fourth, even within interventions targeting all children in a school, inclusion for children with disabilities could be inadequate due to limited adaptations of lessons to allow children with different impairments to participate and school or intervention staff may not be aware of, or address, disability-related barriers to participation. Fifth, interventions may not include content that addresses the specific drivers of violence against children with disabilities, such as disability-targeted discrimination and stigma. Since children with disabilities make up an estimated 1 in 10 children worldwide^37^ and are at high risk of multiple forms of violence,^14^ school interventions aiming to reduce violence are unlikely to be ‘successful’ if children with disabilities are not adequately considered in the intervention or research design.

To date, no review has systematically examined the inclusion and effectiveness of school-based violence prevention interventions assessed in randomised controlled trials (RCTs) for children with disabilities. This review aims to (1) assess the extent that children with disabilities are considered or included in evaluations of school-based violence prevention interventions; (2) synthesise the effectiveness and adaptations of school-based interventions for children with disabilities; (3) highlight gaps in knowledge on disability-inclusive school-based interventions.

## Methods

### Search strategy and selection criteria

A study protocol was registered on PROSPERO (CRD42023463384). Recognising the large number of existing systematic reviews on school violence prevention, we conducted screening in three stages. In the first stage, a systematic search was conducted by the lead author to identify existing systematic reviews of school-based violence prevention interventions. Search terms were created in consultation with a librarian, using Boolean operators, MESH terms, and synonyms of ‘randomised controlled trial’ AND ‘child∗’ AND ‘school’ AND ‘violence’ AND ‘review’. A search strategy can be found in Appendix S1. Records with all of these terms in their title or abstract were retrieved across the following databases: Medline, Cochrane Library, Embase, Global Health, PsycINFO, Web of Science. Searches were conducted in July 2023 and limited to the last 5 years. Searches were not restricted by language, but search terms were in English only. Title and abstract screening was completed first, followed by full text screening. At each stage, screening was conducted by the lead author and 10% of papers (n = 536) were double screened by a second reviewer (AZA). Conflicts at each stage were discussed and resolved between EE and AZA, including n = 6 at title/abstract screening and n = 1 at full text. Screening was conducted using Covidence software. We identified a further 2 systematic reviews through professional networks of colleagues working on school-based violence prevention synthesis in May 2024. One of these was a comprehensive review of school violence interventions to prevent gender-based violence which was unpublished, with searches conducted up to December 2023.

In the second stage, we selected all papers included in the selected systematic reviews and the lead author conducted a further round of full text screening (with 10% double screened by AZA and a discordance of 26% which was discussed and resolved). Here, our approach differs from a typical ‘umbrella review’ as the identified systematic reviews are only used to ‘sample’ our final papers for inclusion. Studies within the reviews were eligible for inclusion if they met all inclusion criteria (following PICOS): (1) randomised controlled trials; (2) interventions delivered in a school-setting to school-attending children; (3) outcomes measured among nursery, primary, and/or secondary school children; (4) violence (outcome). When the authors did not label their studies as an RCT, these were included if they met the criteria of an RCT design: (1) one or more experimental groups receiving treatment; (2) one control group not receiving the treatment; (3) random allocation to treatment and control groups. We also excluded the following articles: theses, conference proceedings, and books. We applied no restrictions to date of publication in this stage. For each included RCT, we searched for any other papers related to this RCT that had conducted a sub-group analysis by searching the intervention or study name into two databases (Google Scholar and PubMed), using the intervention or trial name alongside disab∗ as search terms and included these articles if they met the inclusion criteria.

In the third stage of screening, the lead author searched the full text of included RCTs. We developed and applied four criteria to assess disability inclusion: (1) any effect stratified by disability; (2) any adaptations to intervention design (intervention is targeted or includes adaptations to support participation of children with disabilities); (3) adaptations to research design to better ensure engagement of children with disabilities in the study; (4) any other mention of disability. For criteria 4, we included any papers that included any mention of disability, functional difficulty, or specialist schools in the first instance (e.g., mentioned disability in the introduction or a table footnote). Then, two authors (EE and AB) met to decide if the mention of disability was suitable for inclusion based on if it included empirical results. We conducted further analysis on studies that met at least one of the criteria.

### Data extraction, risk of bias assessment and data analysis

Data was extracted using a pre-specified template, including: citation, study location, violence outcome measure, instrument measuring violence, intervention name, school type, sample size, age range, sex of participants, study design, description of disability and measurement, effect estimate by disability (uncertainty, factors adjusted for, precision). Data extraction was conducted by the lead author, with all papers in the final data extraction sample checked by AZA.

To assess the risk of bias in the trials that included an effect estimate, we used the Cochrane Risk of Bias tool and rated the risk of bias to be ‘low’, ‘some concerns’, or ‘high’.^38^ An overall score was given based on the sum of each domain. To review ethical standards, we included criteria relating to the quality of the research with children and on disability. We considered studies to be higher quality if they: reported a plan to refer children who disclosed violence to services; if they received ethical approval; if they conducted child assent and parental consent; if they reported interviewing children in private, including appropriate child-friendly study procedures. Quality assessment was conducted by the lead author, with the final sample assessed independently by AZA. EE and AZA discussed discrepancies and agreed on the final quality scores.

Meta-analysis was not possible due to the diversity in violence outcomes, and different effect measures. We synthesise the evidence using narrative synthesis by providing structured reporting of the study effects following PRISMA guidance.^39^

### Role of the funding source

The funder had no role in the research design or aims of this review.

## Results

**Fig. 1:**
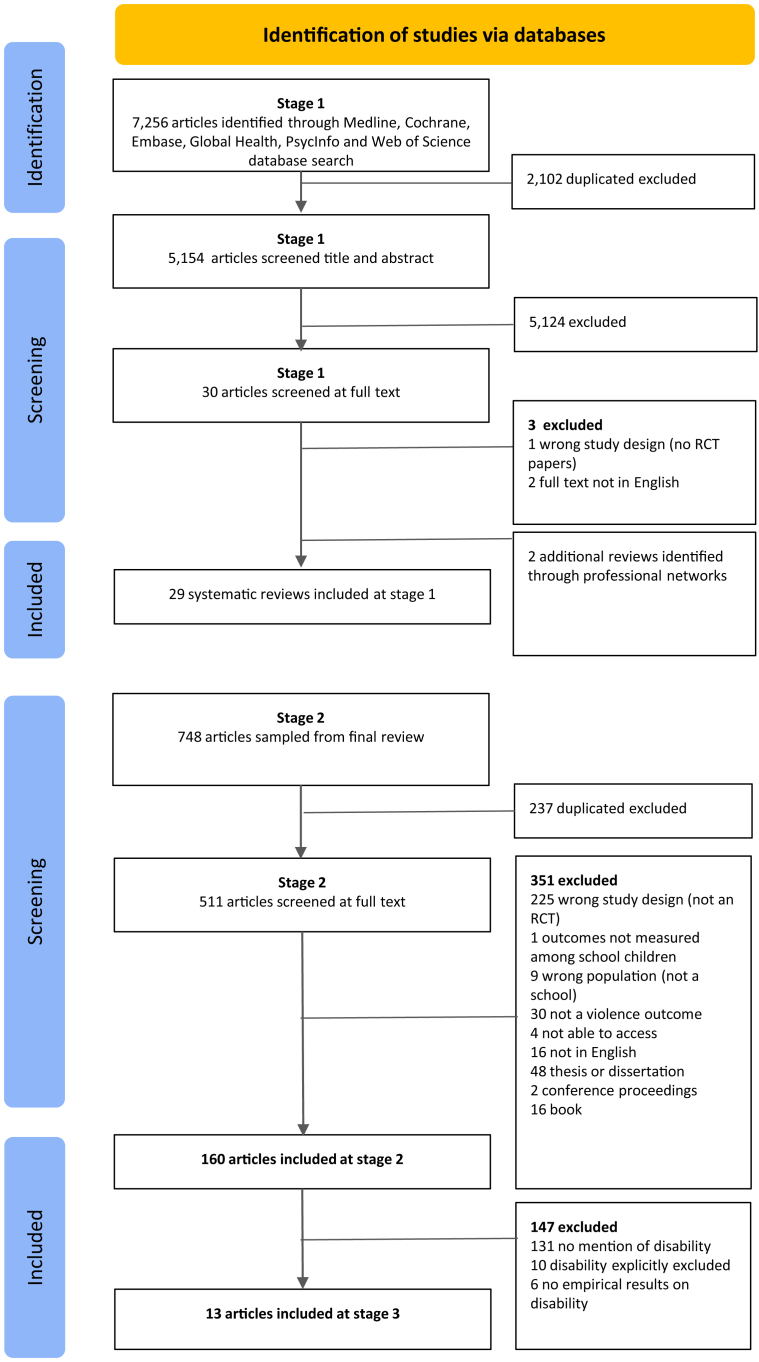
PRISMA.

The initial search returned a total of 5154 articles after duplicates were removed in stage 1 (Fig. 1). After title and abstract and full text screening a total of 29 systematic reviews were identified.^21^, ^22^, ^23^, ^24^, ^25^, ^26^, ^27^, ^28^, ^29^, ^30^, ^31^^,^^40^, ^41^, ^42^, ^43^, ^44^, ^45^, ^46^, ^47^, ^48^, ^49^, ^50^, ^51^, ^52^, ^53^, ^54^, ^55^, ^56^ In stage 2, using these reviews to sample the papers, a total of 511 articles were identified after duplicates were removed. Out of these 511, we identified a sample of 160 papers reporting RCT results of school-based interventions targeting different violence prevention outcomes.

Of these 160 articles, 10 articles (6.25%) explicitly excluded children with disabilities from their study sample, 131 articles (81.88%) did not mention disability, and 6 articles (3.75%) referenced disability but had no empirical results, which included reporting disability as an adverse event (n = 1), mentioning disability affecting standardisation of programme delivery (n = 1) and mentioning disability in the introduction or discussion only (n = 4). This yielded a final sample of 13/160 articles (8.13%) at stage 3, reporting on 10 trials. Table 1 outlines the study characteristics of included studies in more detail.

**Table 1:** Study characteristics of trials including disability.

Trial characteristics							Reference to disability in each trial				
	Lead author, year	Intervention name	Study characteristics (location; school type^a^; sex; age)	RCT design (design; follow up; registration)	Violence outcome (outcome; measurement)	Author description of disability,^b^ measurement	(1) Measure of effect by disability (Y/N)	(2) Disability targeted intervention (Y/N)	(3) Disability adaptations to intervention (Y/N)	(4) Disability adaptations to data collection (Y/N)	(5) Disability status reported in sample^c^ (Y/N)
1	Devries et al., 2018^17^	Good Schools Toolkit	**Location:** Uganda **School type:** primary, mainstream **Sex:** mixed **Age:** 11–14 years	**Design**: cluster randomised controlled trial with parallel assignment, randomisation at school level **Follow up:** 1–2 months after intervention **Trial registration:**clinicaltrials.gov ID: NCT01678846	**Outcome:** any violence from staff in the past week physical **Measure:** ICAST-CI	**Description:** any functional difficulty in the following domains: sight, hearing, movement, memory/concentration, self-care, communication **Measurement:** Washington Group Short Set	Yes	No	No	Yes	Yes
2	Devries et al., 2017^57^										
3	Devries et al., 2015^58^										
4	Lee et al., 1998^59^	Behavioural Skills Training Program	**Location:** Hong Kong **School type:** secondary, specialist **Sex:** female **Age:** 11–15 years	**Design**: individually randomised design **Follow up:** (1) within 1 week; (2) 2-month **Trial registration:** no	**Outcome (1):** ability to differentiate appropriate from inappropriate sexual advances **Measure:** ‘What if’ situation test **Outcome (2):** knowledge about self-protection skills **Measure:** ‘What if’ situation test **Outcome (3):** knowledge of sexual abuse **Measure:** personal safety questionnaire	**Description:** mental retardation [sic] **Measurement:** assessment of qualified educational psychologists prior to admission to the special schools	Yes	Yes	Yes	No	Yes
5	Cissner et al., 2014^60^	Fourth R	**Location:** USA **School type:** secondary, mainstream **Sex:** mixed **Age:** 11–14 years	**Design:** cluster randomised controlled trial, student-level randomisation **Follow up:** (1) end of intervention school year; (2) 1 year after **Trial registration:** no	**Outcome:** school violence victimisation & perpetration **Measure:** Youth Risk Behaviour Survey	**Description:** students with disabilities and special needs **Measurement:** students receiving individualised educational programs	No	No	No	No	Yes
6	Cappella et al., 2012^61^	BRIDGE	**Location:** USA **School type:** primary, mainstream **Sex:** mixed **Age:** 8 years (mean)	**Design**: cluster randomised controlled trial, randomisation at classroom level **Follow up:** post-test **Trial registration:** no	**Outcome:** peer victimisation **Measure:** Social Behaviour and Experience Questionnaire	**Description:** students in special education and combined classes^d^ **Measurement:** not defined	No	No	No	No	Yes
7	Espelage et al., 2016^62^	Second Step: Student Success Through Prevention (SS-SSTP)	**Location:** USA **School type:** secondary, mainstream **Sex:** mixed **Age:** 11–12 years	**Design:** cluster randomised controlled trial, randomisation at school level **Follow up:** (1) post-test; (2) 1 year after intervention **Trial registration:**clinicaltrials.gov ID: NCT01792167	**Outcome (1):** bullying perpetration over the past 30 days **Measure:** Illinois Bully Scale **Outcome (2):** peer victimisation in the past 30 days **Measure:** Illinois victimisation scale **Outcome (3):** physical aggression over the past 30 days **Measure:** not reported **Outcome (4):** willingness to intervene in bullying **Measure:** Illinois willingness to intervene	**Description:** cognitive disability; emotional disability; health impairment; multiple disabilities; specific learning disability; speech/language impairment **Measurement:** student's legal disability diagnosis	Yes	No	No	Yes	Yes
8	Espelage et al., 2015^63^										
9	Holen et al., 2013^64^	Zippy's Friends	**Location:** Norway **School type:** primary, mainstream **Sex:** mixed **Age:** 7–8 years	**Design:** cluster randomised controlled trial with matched pairs assignment, randomisation at school level **Follow up:** immediately after completion of programme **Trial registration:** no	**Outcome:** bullying construct in class climate scale **Measure:** FEESS 1–2: questionnaires on emotional and social experiences of primary school children, first and second grades	**Description:** students receiving special teaching^d^ **Measurement:** not defined	No	No	No	No	Yes
10	Stallard et al., 2013^65^	The Resourceful Adolescent Programme	**Location:** UK **School type:** secondary, mainstream **Sex:** mixed **Age:** 12–16 years	**Design:** three-arm cluster randomised controlled trial, randomised at the school level **Follow up:** 12 months after baseline **Trial registration:** Current Controlled Trials ISRCTN19083628	**Outcome:** bullying perpetration and victimisation **Measure:** Olweus Bully/Victim Questionnaire	**Description:** students with special needs^d^ **Measurement:** not reported	No	No	No	No	Yes
11	Waasdorp et al., 2012^66^	School-wide Positive Behavioural Interventions and Supports (SWPBIS)	**Location:** USA **School type:** primary, mainstream **Sex:** mixed **Age:** kindergarten to 5th grade	**Design:** group randomised controlled effectiveness trial, randomised at school level **Follow up:** timepoint unclear **Trial registration:** no	**Outcome:** teacher-reported bully related behaviour **Measure:** teacher observation classroom adaptation checklist	**Description:** students with special education status^d^ **Measurement:** not reported	No	No	No	No	Yes
12	Snyder et. al., 2013^67^	Positive Action	**Location:** USA **School type:** primary, mainstream **Sex:** mixed **Age:** 5th grade	**Design:** matched pair cluster randomised controlled trial, randomised at school level **Follow up:** one year post trial **Trial registration:**clinicaltrials.gov ID: NCT00328445	**Outcome (1):** student-reported involvement in violent behaviour **Measure:** questions from Monitoring the Future and the Aban Aya Youth Project adapted for this survey **Outcome (2):** teacher reported violence behaviour **Measure:** Risk Behaviour Survey	**Description:** students receiving special education^d^ **Measurement:** not reported	No	No	No	No	Yes
13	Jones et al., 2020^68^	Orbit	**Location:** Australia **School type:** primary, mainstream **Sex:** mixed **Age:** 8–10 years	**Design:** randomised controlled trial, randomisation at classroom **Follow up:** post-intervention (time point unclear) **Trial registration:** no	**Outcome:** knowledge of abuse prevention **Measure:** Children's Knowledge of Abuse Questionnaire-Revised	**Description:** children with disabilities **Measurement:** not reported	No	No	Yes	No	No

a School type includes: nursery, primary or secondary and whether the school is mainstream or specialist.

b We include authors description of disability.

c We include papers that reported inclusion of children with disability in their sample or used disability as a covariate only, for instance disability is mentioned in the participant characteristics.

d We include papers that included classes or children who received special teaching as these are likely to include children with disabilities, though the articles did not provide a definition.

Of the 10 trials that included more substantive mentions of children with disabilities: 3 trials reported on the magnitude of effect of the intervention for children with disabilities^17^^,^^57^, ^58^, ^59^^,^^62^^,^^63^; 4 trials mentioned adaptations to intervention or research design^17^^,^^57^, ^58^, ^59^^,^^62^^,^^63^^,^^68^; 6 trials mentioned disability as part of the baseline characteristics of trial participants but did not include further analysis.^60^^,^^61^^,^^64^, ^65^, ^66^, ^67^

Trials were in Australia (n = 1), Uganda (n = 1), Hong Kong (n = 1), USA (n = 5), Norway (n = 1), and the UK (n = 1). There were more trials based in secondary schools than primary schools. Trial outcomes focused on teacher physical violence (n = 1), sexual abuse (n = 2), and bullying or violent behaviour between peers (n = 7). None of the 45 articles of trials in the 160 sample that focused on IPV/relationship violence or cyberbullying included disability.

Trials used different measures of disability, including clinical screening (n = 1), Washington Group Short Set questionnaire (n = 1), and administrative data (n = 2). 6 trials did not define how disability was measured. Only 2 trials disaggregated descriptive results by type of disability/functional limitation^17^^,^^57^^,^^58^^,^^62^^,^^63^ (Fig. 1).

Out of 10 trials, 2 studies included reporting adaptations to intervention design and 2 reported adaptations to research design for children with disabilities.^17^^,^^57^, ^58^, ^59^^,^^62^^,^^63^^,^^68^ Across the studies, reported adaptations for children with disabilities were minimal. Further details are reported in Appendix S3.

For the intervention adaptations, Orbit (Australia) intervention reported brief adaptations suggesting there was some content that aimed to be more inclusive of disability, including a video game character using a wheelchair. However, there was no reporting on additional examples. Despite the intervention being targeted to children with intellectual disabilities, the Behavioural Skills Training Programme (Hong Kong) mention few moderations to the intervention design, which was originally intended for children without disabilities in the USA, and no adaptations to their data collection procedures. However, the authors suggest adaptations to the design for future study including increasing the length of the sessions and adding general sexual health education to improve overall knowledge.

The Good Schools Toolkit (Uganda) and SS-SSTP (USA) are complex interventions delivered in mainstream schools. Neither intervention was adapted for children with disabilities; however, both studies report some adaptations to the data collection for disability, including training of interviewers in disability adjustments. However, further examples were not provided.

Out of 10 trials, 3 trials included an effect estimate for children with disabilities and we carried out quality assessment on these trials only. Overall, the risk of bias across the 3 trials were varied (Table 2). The Good Schools Toolkit trial (which included 3 papers) received a ‘low’ score overall and the SS-SSTP trial (which included 2 papers) and the Behavioural Skills Training Programme trial received a score of ‘some concerns’. The Behavioural Skills Training Programme score related to poor reporting on several of the domains (e.g., no analysis section in methods, no overall explanation of randomisation method, or information on attrition/missingness in entire sample). Out of 3 trials, only the Good Schools Toolkit trial reported on all child protection considerations in the articles (Table 2).

**Table 2:** Quality assessment of trials reporting effect estimates by disability.

Assessment range for the Cochrane Risk of bias includes green = low risk; orange = some concerns; red = high-risk.

^a^In this section we consider if the following items are reported for both children with and without disabilities: (1) ethical clearance; (2) parental consent and child assent procedure; (3) privacy and safety during interview; (4) referral procedures. Note for trials with sub-group analysis, we reviewed the trial paper for further details.

Only 3/10 trials presented an effect estimate for children with disabilities (Table 3).^17^^,^^57^, ^58^, ^59^^,^^62^^,^^63^ The Good Schools Toolkit intervention was found to decrease violence from teachers to children with disabilities (0.27 OR, 95% CI, 0.13, 0.56) and children with any functional limitations (0.40, 95% CI, 0.23–0.69) in the intervention compared with the control. The trial found no differential effects of the intervention by disability compared to no functional difficulty (LR test p = 0.342), or children without any disability or functional difficulty. In other words, the Good School Toolkit intervention reduced violence in all children, with or without any disability.

**Table 3:** Study outcomes of trials reporting effect estimates.

Intervention name	Total number of participants	Number of participants with disability	Outcome	Analysis and measure of association	Author reported effect estimates	Intervention group	Comparison group	Follow up	Interpretation
Good Schools Toolkit^17^^,^^a^	3820	220	Any violence from staff in the past week	Stratified logistic regression; adjusted OR	Adjusted OR: 0.27, 95% CI (0.13, 0.56)	Students reporting a lot of difficulty or cannot do in one functional difficulty domains in the intervention condition	Students reporting a lot of difficulty or cannot do in one functional difficulty domains in the control condition	Within 1 month	Children with disabilities experienced a decrease in violence from teachers in the intervention group compared to the control group
	3820	644	Any violence from staff in the past week	Stratified logistic regression; adjusted OR	Adjusted OR: 0.40, 95% CI (0.23, 0.69)	Students reporting some difficulty in one functional difficulty domain in the intervention condition	Students reporting some difficulty in one functional difficulty domain in the control condition	Within 1 month	Children with any functional difficulties experienced a decrease in violence from teachers in the intervention group compared to the control group
Behavioural Skills Training Programme^59^^,^^b^	72	72	Overall group by time interaction	MANOVA, group main effect	F (4, 67) = 3.83, p < 0.01	All students with disabilities in the intervention condition	All students with disabilities in the control condition	Within the week after the intervention	Children with disabilities experienced an effect across all pooled outcomes compared to the control condition
			Appropriate request recognition	MANOVA, univariate 2-way interaction effect	F (2, 140) = 4.08, p < 0.05	All students with disabilities in the intervention condition	All students with disabilities in the control condition	Within the week after the intervention	Children with disabilities experienced an increase in recognition of appropriate touch requests in the intervention group compared with the control group
			Knowledge about self-protection skills	MANOVA, univariate 2-way interaction effect	F (2, 140) = 20.48, p < 0.001	All students with disabilities in the intervention condition	All students with disabilities in the control condition	Within the week after the intervention	Children with disabilities experienced an increase in knowledge about self-protection skills in the intervention group compared with the control group
			Knowledge of sexual abuse	MANOVA, univariate 2-way interaction effect	F (2, 140) = 7.37, p < 0.005	All students with disabilities in the intervention condition	All students with disabilities in the control condition	Within the week after the intervention	Children with disabilities experienced an increase in knowledge about sexual abuse in the intervention group compared with the control group
Second Step: Student Success Through Prevention (SS-SSTP)^62^^,^^63^	3658	123	Bullying perpetration over the past 30 days	Linear mixed growth model, time × condition, intervention coefficient	β17 = −0.15 SE-0.07, p < 0.05, 95% CI (−0.28, −0.02)	Any students reporting any disability (according to their legally identified category) in the intervention condition	Any students reporting any disability (according to their legally identified category) in the control condition	Immediately post-test	Children with disabilities decreased bullying perpetration in the intervention group compared with the control group
			Peer victimisation in the past 30 days	Linear mixed growth model, time × condition, intervention coefficient	β17 = −0.04 SE-0.11, p > 0.05, 95% CI (−0.27, 0.18)	Any students reporting any disability (according to their legally identified category) in the intervention condition	Any students reporting any disability (according to their legally identified category) in the control condition	Immediately post-test	No significant interaction effect
			Physical aggression over the past 30 days	Linear mixed growth model, time × condition, intervention coefficient	β17 = −0.13 SE-0.07, p > 0.05, 95% CI (−0.28, 0.02)	Any students reporting any disability (according to their legally identified category) in the intervention condition	Any students reporting any disability (according to their legally identified category) in the control condition	Immediately post-test	No significant interaction effect
			Willingness to intervene in bullying^c^	ANCOVA, adjusted standardised mean-difference effect sizes	g = 0.67 p < 0.05, 95% CI (0.21, 1.14)	Any students reporting any disability (according to their legally identified category) in the intervention condition	Any students reporting any disability (according to their legally identified category) in the control condition	Immediately post-test	Children with disabilities showed an increase in willingness to intervene in bullying incidents in the intervention group compared with the control group

a Only the paper reporting the sub-group analysis for this trial is included here.

b Of the four outcome measures, only figures for the three significant outcomes were provided in the original article.

c Wave 3 was used in accordance with the main trial paper.

The Behavioural Skills Training Programme was found to increase recognition of appropriate touch requests (F (2, 140) = 4.08, p < 0.05), increase knowledge about self-protection skills (F (2, 140) = 20.48, p < 0.001), and sexual abuse (F (2, 140) = 7.37, p < 0.005) among children with intellectual disabilities in the intervention compared to the control. No significant intervention effects were found for recognition of inappropriate touches, which was a distinct outcome from appropriate touch requests. However, these results were not sustainable over time with no significant intervention effects found for the 2-month follow up.^59^

The SS-SSTP intervention was found to decrease bullying perpetration (β = −0.15 SE-0.07, p < 0.05, 95% CI, −0.28, −0.02) and increase willingness to intervene in bullying incidents (g = 0.67 p < 0.05, 95% CI, 0.21, 1.14) amongst children with disabilities in the intervention condition compared with the control condition. The trial did not assess the differential effect of children with disabilities compared to children without disabilities. No significant sub-group effects were found for peer victimisation and physical aggression.

## Discussion

Although childhood disability is common and children with disabilities have an elevated risk of violence, the 160 articles of randomised controlled trials testing school-based violence prevention interventions are virtually silent on issues of disability. Within the sample of 13 articles reporting on 10 trials (8.13%) which even mentioned disability in their trial sample, analysis or intervention adaptations, evidence is largely limited to high-income countries, and mainstream schools which are inclusive of all children. Of 160 articles, 10 articles (6.25%) explicitly excluded children with disabilities. The remaining 137 articles (85.63%) were based in mainstream schools, so they will have included children with disabilities, but studies did not measure disability or engage with disability content in the intervention. This is consistent with other public health studies and previous reviews that find children with disabilities are frequently invisible within trials and other research designs^32^, ^33^, ^34^, ^35^, ^36^^,^^69^ as disability is either not measured, or not included in the analysis if measured.

This review is the first systematic review examining the inclusion and effectiveness of school-based interventions for children with disabilities assessed in RCTs—who are at high-risk of experiencing violence—aiming to provide impetus to researchers and practitioners working in violence prevention to consider disability in design and delivery. Encouragingly, our findings show that when studies examine intervention effects among children with disabilities, interventions can reduce school-based violence among children with disabilities: All 3 trials that reported effect estimates stratified by disability status, found the intervention reduced school-based violence among children with disabilities. However, the risk of bias scores ranged from ‘low’ to ‘some concerns’ with significant concerns relating to the lack of reporting on several of the domains, therefore the results on stratified effect estimates should be taken with some caution. Results from these 3 trials are largely consistent with the main trial analyses inclusive of all children, suggesting that at least some universally targeted interventions can also be effective for children with disabilities.

In the 10 trials that mentioned disability, no trials test interventions to prevent IPV/relationship violence or cyberbullying outcomes amongst children with disabilities despite numerous such interventions targeted at all children.^21^^,^^23^^,^^25^, ^26^, ^27^^,^^48^^,^^49^^,^^52^^,^^53^ This gap further underscores the paucity of engagement with disability we document in this paper. Prior studies^70^^,^^71^ suggest that the lack of evidence on IPV and relationship violence could also be indicative of assumptions about intimate relationships amongst people with disabilities. The lack of intervention in cyberbullying for children with disabilities is noteworthy given recent evidence suggesting children with disabilities experience high levels of cybervictimisation.^14^

Given children with disabilities are a diverse group and have different access needs (e.g., accessible communication, accessible infrastructure, inclusive transport services), it is important that interventions and data collection procedures are designed appropriately. We find adaptations to intervention and data collection designs for children with disabilities, with attention to types of impairments, are underreported across the trials and only reported by 4 of the trials. Several frameworks exist on adapting interventions, including the GRAIDs framework—Guidelines, Recommendations, Adaptation Including Disability—for practitioners, researchers and government agencies creating accessible health promotion programmes,^72^ and on creating accessible research design that focuses on creating accommodations in the setting, study tools, participant responses, scheduling, and timing.^73^ Yet, no comprehensive guidelines exist for creating adapted intervention or research design, inclusive of different disabilities, of school-based interventions within violence prevention specifically.

There are several limitations to our approach. Firstly, our review focuses only on RCTs in order to assess the ‘gold-standard’ evidence globally and to examine effect estimates. However, RCTs are expensive and time-consuming to implement, and there may be several inclusive interventions evaluated under different study designs, such as quasi-experimental or qualitative design.^74^^,^^75^ Secondly, papers were limited to English language. Thirdly, due to our use of systematic reviews to sample the papers, it is possible that we may have missed some papers in searches and screening, however the most recent systematic review included papers up to December 2023.

Our review aimed to provide evidence on a broad range of violence prevention interventions, however, the sample of the papers that mentioned disability only included trials of bullying, sexual violence, and teacher violence, therefore conclusions cannot be drawn on other violence outcomes such as dating violence or cyberbullying. Since the violence outcomes and effect measures were diverse, and the sample was small, it was not possible to conduct a meta-analysis or quantitative synthesis, and we therefore could not provide a pooled estimate on intervention effects for children with disabilities. In several of the trials included in the final sample (n = 10), disability lacked specificity in terms how it was defined and measured. For instance, trials reported the inclusion of children receiving special education in their sample without defining which children were included in this group or how this was measured.

This review has key implications for research and programming. Researchers conducting disability-inclusive trials should train data collection teams on disability, adapt data collection for different impairments (e.g., providing visual aids or sign-language interpreters), provide alternative assent procedures (e.g., thumbprint or witness signature).^76^ Future RCT research should also consider oversampling and powering trials to enable sub-group analysis by disability to understand differences in intervention effects for children with and without disabilities. This analysis should be considered alongside other sociodemographic characteristics such as sex, age, ethnicity, sexuality, and income status (Eldred, forthcoming). Without such analysis, it will remain unclear whether ‘successful’ violence prevention interventions extend their effectiveness to all children, especially those who may be at higher risk of violence victimisation. Within violence prevention specifically, research should be conducted, and reported, in line with ethical child protection procedures—including adaptations on referral procedures for children with disabilities—to ensure the safety of participants^77^ and disability-targeted violence should also be measured. Future reviews on this topic could include search terms and articles in non-English language to identify a larger sample of papers.

Practitioners designing and delivering school-based violence prevention interventions should consider including accessible content and communication (e.g., Braille, simplified-language booklets, or augmentative and alternative communication), inclusive media or promotion materials (e.g., drawings of children with disabilities in posters or booklets), improving school building design (e.g., ramps to building), training for content delivery personnel (e.g., training for teachers and students on different disabilities), adaptations to target additional drivers of violence against children with disabilities (e.g., reducing stigma through education or contact-based interventions^78^^,^^79^) and engaging with local Organisations of Persons with Disabilities to tailor intervention design. We note that the KiVa intervention, an evidence-based anti-bullying intervention originating in Finland, is piloting adaptations to the intervention to special schools in the UK,^80^ including one school for children with autism and the other for severe and complex learning disabilities. The adaptations were made in consultation with specialist staff and included simplifying text and additional handouts with pictures alongside text.^80^ Guidelines, co-created with children with disabilities and school stakeholders, on conducting disability-inclusive violence prevention interventions within schools would be valuable. Most interventions reviewed in this paper are implemented within mainstream schools, targeting all children. While these interventions may reduce some forms of violence for children with disabilities, bespoke interventions may also be needed to address the specific disability-targeted forms of violence and tailored programming relating to specific impairments. For instance, children with intellectual or communication disabilities can face barriers in reporting violence to child protection services, and programming that includes additional accessible reporting mechanisms are needed.^81^ This combination of mainstream and tailored interventions is often called a ‘twin-track’ approach, which is useful for ensuring that all school-going children with disabilities benefit from violence prevention efforts. Importantly, although our findings relate to school-based violence programmes and corresponding outcomes, children with disabilities should be included in all violence prevention interventions.

Within public health research and practice, we still know relatively little about the effectiveness of school-based interventions for children with disabilities and how current interventions can be adapted to be more inclusive. To meet universal targets such as the Sustainable Development Goals (SDGs) and meet the needs of children with disabilities, school-based violence prevention interventions should design targeted interventions and adapt current interventions for children with disabilities, and evaluations should be designed to test if interventions work for children with disabilities, especially before interventions are scaled.

## Contributors

EE, KD, LMB, and AB conceptualised this work. EE conducted the searches, screening and data extraction, prepared the tables and figures, and drafted the manuscript. AZA and AB contributed to the screening and data extraction. All authors commented on and/or edited the final manuscript and had access to the data included in this study. All authors approved the final manuscript.

## Data sharing statement

All data included in this review are publicly available online.

## Declaration of interests

We declare no competing interests.
