# Supplementary material for: Are school-based violence prevention interventions inclusive and effective for children with disabilities? A systematic review of global evidence
Source: eClinicalMedicine. 2025 Jan 17;80:103060. doi: 10.1016/j.eclinm.2024.103060 (PMC11787422; doi:10.1016/j.eclinm.2024.103060)
Supplement: Appendix S1–S3 [file mmc1.docx]

**Appendix 1. Search Terms & Databases**

Databases to search: MEDLINE, EMBASE, PsycINFO, Global Health, and Web of Science (Science and Social Science Citation Index); Cochrane Library

OVID

Medline (1699), Embase (2375)

1. Randomized Controlled Trial/ or Early Intervention, Educational/

2. (prevention or preventing or reduce or reducing or intervention or programme or program or project* or initiative or evaluation or evaluate or trial or randomi#ed control trial or effectiveness). ti,ab,kw.

3. 1 or 2

4. Child/ or Adolescent/ or Young Adult/ or Infant/ or Infant, Newborn/ or Students/ or Child, Preschool/

5. (child* or adolescen* or boy$1 or boyhood or girl* or teen* or preteen* or pubescen* or prepubescen* or youth* or juvenile* or preteen* or pre teen* or young people* or young person* or early adult* or young adult* or infan* or baby or babies or school* or pupil* or student* or nursery or preschool* or pre school* or underage or minor or partner* or spouse* or peer* or boyfriend* or boy friend* or girlfriend* or girl friend or acquaintance* or non stranger* or nonstranger* or agemate or age mate).ti,ab,kw.

6. 4 OR 5

7. Schools, Nursery/

8. (School* or classroom or learning institution or educational institution or school-based or education). ti,ab,kw.

9. 7 OR 8

10. Violence/ or Physical Abuse/ or Rape/ or Adverse Childhood Experiences/ or Child Abuse/

11. (violence or violent or aggression or maltreatment or maltreat* or abuse or corporal punish* or discipline or spanking or caning or peer violence or peer relationships or bully* or bullied or anti bully* or bully victim or cyberbull* or cybervictim* or intimate partner violence or IPV or gender based violence or GBV or dating or teen relationships or violence against children or VAC or coercion or defilement or rape$1 or rapist or incest or polyvictim* or pedophil* or peadophil* or sexual assault or restraining or exclusion or neglect* or psychosocial). ti,ab,kw.

12. sex* adj2 (violen* OR abus* OR assault* OR attack* OR aggressi* OR coerc* OR maltreat* OR victim* OR re victim* OR offence* OR offense* OR molest* OR harass* OR exploit*).ti,ab,kw

13. phys* adj2 (violen* OR abus* OR assault* OR attack* OR aggressi* OR coerc* OR maltreat* OR victim*).ti,ab,kw.

14. ((emotion* OR psychologic* OR mental) adj2 (violen* OR abus* OR maltreat*)).ti,ab,kw.

15. ((gender or peer) adj2 (violen* OR abus* OR assault* OR attack* OR aggressi* OR coerc* OR maltreat* OR victim* OR harass*)).ti,ab,kw.

16. ((intimate partner OR domestic partner OR partner* OR relationship* OR spouse* OR boyfriend* OR boy friend* OR girlfriend* OR girl friend OR date OR dating OR acquaintance* OR non stranger* OR nonstranger*) adj2 (violen* OR abus* OR assault* OR attack* OR aggressi* OR coerc* OR maltreat* OR victim*)).ti,ab,kw.

17. ((witness* OR expos*) adj2 (violen* OR abus* OR assault* OR attack* OR aggressi* OR coerc* OR maltreat* OR harass*)).ti,ab,kw.

18. 10 or 11 or 12 or 13 or 14 or 15 or 16 or 17

19. Review/ or Systematic Review/ or meta-analysis/

20. (Review* or systematic review or meta-analy* or meta analy* or synthesis) or (((comprehensive* or integrative or systematic*) adj3 (bibliographic* or review* or literature)) or (meta-analy* or metaanaly* or "research synthesis" or ((information or data) adj3 synthesis) or (data adj2 extract*))).ti,ab,kw.

21. 19 or 20

22. 3 and 6 and 9 and 18 and 21

23 limit 22 to last 5 years

**Appendix 2. Reasons for exclusion at full text (phase 2)**

| **Reason** | **Number** |
| --- | --- |
| Wrong study design (not a randomised control trial) | 225 |
| Primary outcomes not measured among nursery, primary and secondary school children | 1 |
| Wrong population (not a school) | 9 |
| Not violence outcome | 30 |
| Not able to access | 4 |
| Studies not in English language | 16 |
| Thesis/dissertation | 48 |
| Conference proceedings | 2 |
| Book | 16 |

**Appendix 3. Adaptations to intervention or research design in school-based violence prevention trials**

|  | **Intervention name** | **Description of intervention** | **Delivery** | **Duration** | **Reported adaptations to intervention design for disability** | **Reported adaptations to data collection design for disability** |
| --- | --- | --- | --- | --- | --- | --- |
| 1 | Good Schools Toolkit (mainstream intervention) | A complex behavioural intervention aiming to target a reduction in violence at the school level | Raising Voices staff train head teachers and ‘protagonist’ teachers to deliver school level activities. These activities are coordinated by two ‘protagonist’ teachers and two student representatives in every school, with monthly support visits from Raising Voices staff | 18 months | None reported | Training for interviewers on adjustments for students with sight, hearing, and other functional difficulties |
| 2 | Behavioural Skills Training Programme  (targeted intervention) | A self-protection skills programme adapted for use in Hong Kong for children with intellectual disabilities (originally designed for USA, Wurtle 1986; 1992) focusing on appropriate and inappropriate touches, body safety, and skills for self-protection, including reporting of abuse | First author delivered two sessions | Two 45-minute sessions | Visual aids used to accompany narrative scripts in teaching sessions | None reported |
| 3 | Fourth R (mainstream intervention) | A 21-lesson skills-based curriculum for adolescents, aiming to develop healthy decision-making in relationships, sexuality and drug and alcohol use | Fourth R intervention staff train teachers in a 6-hour workshop to deliver the curriculum to students | 21 75-minute lessons | None reported | None reported |
| 4 | BRIDGE (mainstream intervention) | A consultation and coaching programme utilising mental health professionals within schools. Teachers and mental health professionals are guided by a tool to understand effective classrooms (the Classroom Assessment Scoring System). Mental health professionals undergo training to work with teachers on classroom interactions and student behaviour | School-based mental health professionals are trained to support teachers | Not reported | None reported | None reported |
| 5 | Second Step: Student Success Through Prevention (SS-SSTP) (mainstream intervention) | A curriculum-based intervention designed for 6^th^ to 8^th^ grade students focusing on social emotional learning (including empathy, communication skills, problem-solving, bullying prevention) with progressive difficulty across the three grades | Teachers trained to deliver curriculum | 15 lessons in 6^th^ grade and 13 in 7^th^ and 8^th^ grade. Lessons are either 50-minutes or two 25-minute lessons | None reported | The data collection was conducted in familiar self-contained classrooms, and the data collection team explained concepts when necessary |
| 6 | Zippy’s Friends (mainstream intervention) | A curriculum-based intervention based on 6 stories relating to family and friends of a cartoon stick insect, Zippy, exploring emotions, communication, relationships and conflict resolution. The programme also includes class discussions, role-playing, drawing, play and performance exercises about the stories | An NGO, Partnership for Children, partners with local organisations in each country to manage the delivery of the programme | 24 weekly lessons | None reported | None reported |
| 7 | The Resourceful Adolescent Programme (mainstream intervention) | Classroom-based cognitive behavioural therapy intervention | Two facilitators external to schools deliver to whole classes | 9 weekly or fortnightly sessions | None reported | None reported |
| 8 | School-wide Positive Behavioral Interventions and Supports (SWPBIS) (mainstream intervention) | The intervention model draws on behavioural, social learning and organisation principals, aiming to alter the school environment through: improved systems (e.g. discipline) and procedures (referral and behaviour reinforcement) to promote changes in staff and student behaviour | The SWPBIS intervention team coordinate the programme, and train 5-6 school staff per school in a 2-day training programme. There is also a 2-day booster session every year. A trained behaviour support coach provides support to schools for the duration of the intervention, who also receive professional development and technical assistance support 4 times per year | 4 years | None reported | None reported |
| 9 | Positive Action (mainstream intervention) | A school-wide programme aiming to influence multiple behaviours, including academic achievement and substance misuse. The intervention includes: teacher and staff training, a coordination manual, a school counsellor programme, a coordinator guide, and family and community based components | Teachers and school staff are trained by the programme developer, for 3-4 hours in year 1 and then 1-2 hours in the following years. Booster sessions are delivered by the project coordinator for 30 to 50 minutes every academic year | 140 lessons per year for each grade in 15–20-minute lesson slots | None reported | None reported |
| 10 | Orbit (mainstream intervention) | Computer game programme addressing child sexual abuse prevention with game elements and classroom activities | Teachers facilitated the game play in class and discussion | 5 to 10 weeks with 5 ‘chapters’ of 1hour 20-2 hours and 20–40-minute discussion | Computer game included a customised computer character to use a wheelchair | None reported |
|  | **Appendix 3. Intervention description and adaptations to intervention or research design** | | | | | |
